# Kawasaki Disease and Coronary Artery Involvement: A Narrative Review

**DOI:** 10.7759/cureus.28358

**Published:** 2022-08-24

**Authors:** Kruthiga Rajasekaran, Shrimahitha Duraiyarasan, Mayowa Adefuye, Nisha Manjunatha, Vinutna Ganduri

**Affiliations:** 1 Research, Rajah Muthiah Medical College & Hospital, Chidambaram, IND; 2 Research, K.A.P. Viswanatham Government Medical College, Tiruchirappalli, IND; 3 Research, University of Ibadan College of Medicine, Ibadan, NGA; 4 Research, Our Lady of Fatima University College of Medicine, Valenzuela, PHL; 5 Research, Bhaskar Medical College, Hyderabad, IND

**Keywords:** aspirin, corticosteroids, kawasaki disease, arteritis, pseudoanurysms, coronary artery

## Abstract

Kawasaki disease is a systemic vasculitis with a risk of developing coronary artery lesions if left untreated. Kawasaki disease can be diagnosed clinically with classical symptoms (conjunctivitis, rash, lymphadenopathy, mucositis, edema of hands and feet), but predicting the risk of developing coronary artery aneurysm remains challenging. The coronary sequelae of Kawasaki disease have significant morbidity and mortality and are the second most common cause of acquired cardiac disease in children. Several genetic and immune factors are involved in the inflammation of coronary artery lesions in Kawasaki disease. Inositol trisphosphate 3-Kinase (ITPKC), Foxp3+, circular RNAs, mannose-binding lectin 2 (MBL2), complement factor H (CFH), kininogen 1 (KNG1), serpin family C member 1 (SERPINC1) and fibronectin 1 (FN1) are the essential genes identified in the pathogenesis of coronary artery lesions in Kawasaki disease. The addition of methylprednisolone to a combination of aspirin and intravenous immunoglobulins and biological agents like anakinra, etanercept, infliximab, and immunosuppressants like cyclosporine prevents the occurrence of coronary artery aneurysms in Kawasaki disease. Since the coronary artery lesions form the second most common cause of acquired cardiac disease in children and the incidence of myocardial infarction is a late complication, the risk stratification for coronary artery aneurysms and follow-up protocols for the prevention of cardiac thrombosis were proposed by the American Heart Association in 2017.

## Introduction and background

Kawasaki disease, also known as acute febrile mucocutaneous syndrome, is a multi-system inflammatory disorder of the blood vessels that mainly affects infants and children under five years of age [1]. In January 1961, Tomisaku Kawasaki, a Japanese pediatrician, encountered his first case of pediatric vasculitis, and in 1967 he published a study in Japanese with over 50 patients suffering from this syndrome [2]. It is more prevalent in Japan, with an incidence reported of over 200/100000, which is much higher than in Western countries, where it is 40/100000. Kawasaki disease varies significantly by race, with an average incidence rate of over 360 for Japanese, 95 for Chinese, 77 for Hawaiians, 56 for Filipinos, and seven for Caucasians. These findings show that racial characteristics, rather than regional considerations, influence vulnerability to Kawasaki disease [3]. In children under five, 80% of Kawasaki cases are reported, while adults account for only 0.7% [4]. The disease has a male predominance with a ratio of 1.5:1 [4,5]. In general, the etiology of Kawasaki disease is unknown, but some studies suggest that the hygiene hypothesis could be a risk factor. Sterile environments, frequent use of antibiotics, sanitizing agents, and formula feeds lead to defective B-cell maturation and low production of immunoglobulins. Dysregulated B cell maturation and differentiation make non-pathogenic triggers pathogenic. This supports the role of immunoglobulins in the treatment of Kawasaki disease [6]. Some recent studies have stated that the co-expression of five immune genes is involved in the development of Kawasaki disease.

Using the Gene Expression Omnibus Database, Kawasaki disease-related databases (GSE18606, GSE68004, GSE73641) were obtained and integrated with samples from 173 Kawasaki patients and 101 samples from regular patients. With the help of CIBERSORT, 22 immune cells were identified in the samples and subjected to differentially expressed gene (DEG) analysis and weighted gene co-expression network analysis (WGCNA). These co-expression networks and Cytoscape 39's cytoHubba tool helped identify these (CXCL8, CCL5, CCR7, CXCR3, and CCR1) genes that play an important role in the pathogenesis of Kawasaki disease [7]. The classic symptoms of Kawasaki disease include fever lasting more than five days, bilateral non-purulent conjunctivitis, dysmorphic skin rashes, edema of hands and feet, and cervical lymphadenopathy and mucositis. Diagnosis is mainly clinical, with a fever lasting more than five days and any four of the five aforementioned clinical criteria [8]. Patients who do not have classic symptoms but have a fever lasting more than five days and evidence of systemic inflammation such as hypoalbuminemia, elevated platelet count, increased WBC count, aminotransferase elevation, and anemia should have an echocardiogram [9]. There are serum biomarkers expressed during acute attacks of Kawasaki disease. Lipopolysaccharide binding protein, leucine-rich 2 glycoprotein, and angiotensinogen are examples of these [10]. The mainstay of treatment for Kawasaki disease is aspirin and intravenous immunoglobulins (IVIG), but recent studies have demonstrated the efficacy of corticosteroids plus IVIG and anti-tumor necrosis factor alpha (TNF-α) drugs in managing IVIG-resistant Kawasaki disease [11]. The serious complication of Kawasaki disease is the involvement of the cardiovascular system. These include pericarditis, myocarditis, coronary artery dilatations, and coronary artery aneurysms. These cardiac lesions are the second most common cause of acquired cardiac disease in children [12]. This review article highlights the association between Kawasaki disease and coronary artery lesions by discussing the risk factors and pathophysiology involved in the development of coronary artery lesions and recent advances in treatment modalities to prevent the development of coronary artery lesions as a consequence of Kawasaki disease.

## Review

Pathogenesis of coronary artery involvement in Kawasaki disease

The pathogenesis of Kawasaki disease remains unclear. Several studies were conducted to determine the theory behind the development of Kawasaki disease based on genetic, immunologic, and infectious factors. Several studies found several associations between genetic and immunologic components in an attempt to identify the pathogenesis of coronary artery involvement in Kawasaki disease. The Japan Ministry of Health first proposed the definition of coronary artery aneurysm, defined as the internal diameter of the lumen of the coronary artery more than 3mm in children less than five years old or more than 4mm in children more than five years old [13].

Interplay between immune and genetic factors

Coronary artery aneurysms and coronary artery disease are two major complications of Kawasaki disease. Using isobaric tags for relative and absolute quantitation (iTRAQ) analysis and western blot, five specific proteins were identified, which include mannose-binding lectin 2 (MBL2), complement factor H (CFH), kininogen 1 (KNG1), serpin family C member 1 (SERPINC1) and fibronectin 1 (FN1). Complement factor H, a negative regulator of C3b protein, and mannose-binding lectin 2 regulate cleavage of C2 and C4 proteins. Low complement factor H and mannose binding lectin 2 increase C3b levels and decrease C2 and C4 separation, causing innate inflammation and contributing to coronary artery damage and thrombosis. In contrast with coronary artery disease (CAD), coronary artery aneurysms (CAA) with KNG1 with histidine proline glycoprotein and SERPINC1 have anti-inflammatory and anticoagulant properties, and their low levels cause inflammation and endothelial damage resulting in coronary artery dilatation and aneurysm formation. Fibronectin 1 is an extracellular glycoprotein involved in wound healing and wound processing. Low levels of fibronectin 1 in CAD and CAA are found [14-18].

Transforming growth factor-beta is one such gene that plays a vital role in T cell activation and cardiac remodeling in Kawasaki disease [19]. Transcription growth factor-beta (TGF-beta) is involved in endothelial cell proliferation, migration, apoptosis, angiogenesis, calcification, and fibrosis. The active and latent form of TGF-beta is regulated by two proteins, FURIN and EMILIN (elastin microfibril interfacer-1). The active form of TGF-beta binds to the TGFBR2 receptor, which recruits TGFBR1 or ACVRL1 (activin type 2 like 1), which further phosphorylates the SMAD family of proteins translocates to the nucleus and causes transcription of TGF-beta. Alteration in TGFB2, TGFBR2, and SMAD3 genes causes continuous transcription and remodeling of coronary endothelial cells and coronary artery aneurysm formation [20].

**Figure 1:**
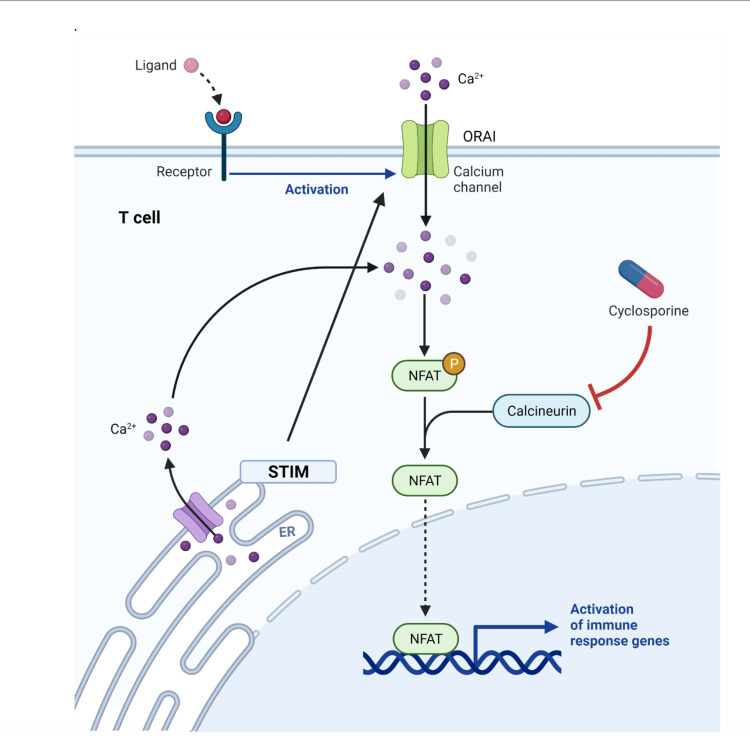
Calcium-dependent NFAT signaling in regulatory T cells. ITPKC gene is a negative regulator of NFAT signaling. Mutated ITPKC causes overaction of NFAT and massive release of inflammatory cytokines. Image Credits: Kruthiga Rajasekaran NFAT: nuclear factor of activated Tcells, ITPKC: inositol trisphosphate 3-Kinase, STIM: stromal-interacting molecule

The most critical gene in the development of coronary artery lesions in Kawasaki disease is the discovery of the inositol 1,4,5-trisphosphate 3-kinase (ITPKC) gene. ITPKC is an important negative regulator of calcium channels and regulates T cells activation. Genetic polymorphisms of ITPKC gene affect its mRNA splicing and cause hyper-activation of calcium channels. The mutation ITPKC3G/C affects premature mRNA splicing, translating into an immature and truncated protein. When an antigen binds to the T cell receptor, activation of phospholipase-gamma one results in hydrolysis of phosphatidylinositol 3,4 bisphosphate to IP3 and DAG. Low activity of the mutated ITPKC gene in Kawasaki disease causes continuous phosphorylation of IP3, which binds to the IP3 receptor in the endoplasmic reticulum and causes calcium release into the cytoplasm. The depletion of calcium in the endoplasmic reticulum is sensed by the stromal interaction molecule (STIM), and the empty endoplasmic reticulum also evokes the store-operated calcium entry (SOC) a process through which extracellular calcium enters via ORAI, a calcium-release activated calcium channel located on the plasma membrane. The cytoplasmic calcium binds to calmodulin, and the calcium calmodulin complex results in the activation of calcineurin, which dephosphorylates the nuclear factor of activated T cells (NFAT), results in continuous transcription of genes involved in T cell activation and release of a large number of inflammatory cytokines which mediates inflammation of coronary artery and formation of aneurysms (Figure 1) [21].

Several proteins are involved in the pathogenesis of Kawasaki disease. N-terminal prohormone of brain natriuretic peptide (NT-pro-BNP) is the potential biomarker of myocardial damage that occurred as a sequela of Kawasaki disease [22]. Cardiac troponin and periostin are non-specific markers elevated in the acute stages of Kawasaki disease [23,24]. Tenascin-C is an extracellular glycoprotein upregulated and used to predict the risk of the development of coronary artery aneurysms [25].

**Figure 2:**
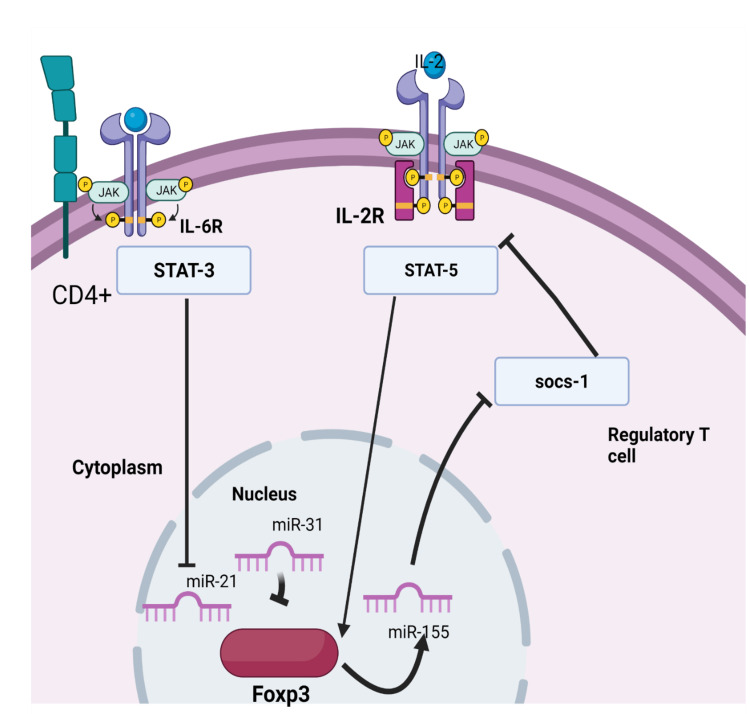
Expression of Foxp3 gene in regulatory T cells. miR-155, miR-31, and miR-21. Foxp3+ directly activates miR-155, which binds to suppressors of cytokines signaling (SOCS)-1 mRNA, thereby inhibiting cytokine signaling. It also indirectly regulates signal transducer and activator of transcription (STAT)-5, which binds to the promoter region of Foxp3+ to increase its expression. miR-31 negatively regulates Foxp3+ expression. miR-21 positively regulates Foxp3+ expression (Figure 1). Interleukin-6, when it binds to its receptor on Treg cells, activates signal transducer and activator of transcription (STAT)-3, which suppresses miR-21. In patients with Kawasaki disease, it is found that low levels of miR-155 and low numbers of Foxp3+ Treg cells and elevated levels of miR-31 and IL-6 are present. Image Credits: Kruthiga Rajasekaran

Another study proposed the role of regulatory T cells (Tregs) cells in the pathogenesis of Kawasaki disease. CD4+CD25+forkhead box protein 3 (Foxp3+) Tregs are crucial in maintaining immune tolerance. Foxp3 is a transcription factor important for developing Tregs. There are three microRNAs (miRNA) involved in the expression of the FoxP3+ transcription factor. These include miR-155, miR-31, and miR-21. Foxp3+ directly activates miR-155, which binds to suppressors of cytokines signaling (SOCS)-1 mRNA, thereby inhibiting cytokine signaling. It also indirectly regulates signal transducer and activator of transcription (STAT)-5, which binds to the promoter region of Foxp3+ to increase its expression. miR-31 negatively regulates Foxp3+ expression. miR-21 positively regulates Foxp3+ expression (Figure 2).

Interleukin-6, when bound to its receptor on Treg cells, activates signal transducer and activator of transcription (STAT)-3, which suppresses miR-21. In patients with Kawasaki disease, it is found that low levels of miR-155 and low numbers of Foxp3+ Treg cells, and elevated levels of miR-31 and IL-6 are present [26-29]. Another miRNA (miR-223) plays a role in forming coronary artery aneurysms in Kawasaki disease. Damage to the endothelium of coronary vessels leads to activation of platelets. miR-223 is not native to vascular smooth muscle cells, but the activated platelets deliver miR-223 to vascular smooth muscle cells, which suppress their de-differentiation through platelet-derived growth factor-beta inhibition. Blood samples of platelet from Kawasaki disease patients with coronary artery aneurysms cultured in vitro show low levels of miR-223, causing massive release of matrix metalloproteinases 9, which damages coronary artery endothelium dilatation and leads to aneurysm formation [30].

Homeodomain interacting protein kinase 3 (circHIPK3), zinc finger protein 124 (circZNF124), WAS protein homolog associated with actin, Golgi membranes, and microtubules pseudogene 1 (circWHAMMP1), SLAIN motif family, member 2 (circSLAIN2), and ataxia telangiectasia mutated (circ ATM) are the circulatory RNAs expressed low in the coronary arteries of untreated Kawasaki disease patients [31]. Urine biomarkers include filamin, talin, complement regulator CSMD3, immune pattern recognition receptor mucin, and immune cytokine protease meprin A, all elevated in acute stages of Kawasaki disease [32].

Histopathology of coronary artery in Kawasaki disease

The histopathology of coronary artery lesions was studied in 37 Japanese patients. The arteries studied include the aorta, carotid, celiac, iliac, hepatic, splenic, mesenteric, renal, lumbar arteries, and the venous system. There are six stages of progressive histopathological findings, including degeneration of endothelial cells, edema and degeneration of the media, necrotizing pan arteritis, granuloma formation, scar formation, and aneurysm formation [33]. There is also an occurrence of myocardial fibrosis in Kawasaki disease patients. Since biopsy of the myocardial tissue is practically difficult, a recent innovation called cardiac integrated backscatter analysis is used in identifying myocardial fibrosis [34].

Diagnosis

Since the first case of Kawasaki disease was reported by Dr. Tamisaku Kawasaki, the criteria for diagnosis remain clinical. There are many criteria proposed in different years for the diagnosis of Kawasaki disease. Based on Japanese guidelines, in 2002, according to the Kawasaki disease research committee, the diagnosis of Kawasaki disease is made when five of the following six symptoms present: persistent fever lasting more than five days, bilateral conjunctival congestion, mucosal involvement, polymorphous eruptions, peripheral extremities involvement, and acute non-purulent cervical lymphadenopathy [35]. In 2004, The American Heart Association proposed its guidelines. These include fever for five days with any four of the five clinical features (erythema of palms and soles, diffuse polymorphic eruptions, bilateral nonexudative conjunctivitis, mucosal involvement, cervical lymphadenopathy) [9].

Echocardiography plays an important role in identifying cardiac complications in Kawasaki disease. The Japanese Ministry of Health criteria first proposed the initial echocardiographic criteria in 2004, which was modified in 2008, which classified aneurysms as small aneurysms, medium aneurysms, and large aneurysms [36]. In 2017 the American Heart Association classified aneurysms with the help of Z scores (Table 1) [37].

**Table 1:** Classification of coronary artery aneurysms in Kawasaki disease AHA: American Heart Association.

2017 AHA classification of coronary artery aneurysms with Z score	2008 modified Japan Ministry of Health criteria
Small aneurysm: ≥2.5 Z score<5	Small aneurysm: ≤4mm internal diameter of coronary artery
Medium aneurysm:≥5 Z score<10	Medium aneurysm:>4mm to ≤8mm internal diameter of coronary artery
Large aneurysm: Z score≥10	Large aneurysm:>8mm internal diameter of coronary artery

Management

Standard Therapy

Aspirin and intravenous immunoglobulins are two drugs conventionally used to treat Kawasaki disease. The initial treatment for Kawasaki disease combines aminosalicylic acid (ASA) with intravenous immunoglobulins [38]. The most important part of treatment in Kawasaki disease is early detection and prevention of cardiac complications, especially coronary artery aneurysms. Several scoring systems were developed to detect the risk of developing coronary artery aneurysms. The Harada scoring system was the first scoring system used to detect coronary artery aneurysms. The factors included in the scoring system are white blood cell count 12,000/mm3, platelet count 350,000/mm3, C-reactive protein, hematocrit 35%, albumin 3.5 g/dL, age ≤ 12 months and male sex. Suppose the child has any four of the fore-mentioned laboratory values within 10 days of illness. In that case, the patient is at risk of developing coronary artery lesions and an indication for immunoglobulins administration [39]. The administration of intravenous immunoglobulins within 10 days of onset of the disease can reduce the risk of the development of coronary artery aneurysms [40]. IVIG suppresses cytokine production, inflammatory markers like CD40L and iNOS, and the provision of anti-idiotypic antibodies [41].

During the initial stages of Kawasaki disease, aspirin should be administered in a high dose (80mg/kg/day) divided every six hours. High-dose aspirin is continued for 14 days during the initial phase of the disease and the child should be fever-free for 48 to 72 hours before stopping high-dose aspirin [42,43]. This high dose of aspirin is followed by a low dose of aspirin (3 to 5 mg/kg daily) for its antiplatelet action and to prevent cardiac complications [44]. While on aspirin therapy, the child should be monitored for the development of Reye syndrome, especially with high-dose aspirin for a prolonged duration of time [45]. Children at risk of developing resistance to intravenous immunoglobulin treatment are treated with corticosteroids as adjunctive therapy. A meta-analysis, including studies, found that the addition of intravenous methylprednisolone to the standard regimen of Kawasaki disease benefits in preventing coronary artery aneurysms development [46].

Biological Agents

Anakinra, an IL-1 antagonist, has been used to manage Kawasaki disease. Several case reports mentioned the introduction of anakinra after the development of coronary artery lesions in Kawasaki disease (Table 2). Guillaume et al. reported a case in 2018 in which an 18-year-old boy with typical Kawasaki disease was treated with IVIG on the sixth day of illness. Methylprednisolone and second dose of IVIG were started after he developed aneurysms on the ninth day of illness; Fever subsided, but aneurysms worsened on the 25th day, and hence anakinra was started on the 25th day of illness. The patient was started on 6mg/kg/day for 10 weeks, reduced to every three days for four weeks with a dose of 6mg/kg/day, and till withdrawal, the patient was administered with 6mg/kg/day but every two days for four weeks. Reduction in C-reactive protein levels and decrease in size of aneurysms after treated with anakinra for 18 weeks as pulse therapy [47].

**Table 2:** Case reports of Kawasaki disease treated with anakinra after developing coronary artery lesions IVIG: intravenous Immunoglobulins

Author	History of patients who developed coronary artery lesions in Kawasaki disease	Treatment before starting anakinra
Guillame et al. [47]	18-month-old boy with typical Kawasaki disease who developed diffuse and fusiform coronary artery aneurysms after seventh day of admission.	IVIG, three boluses of corticosteroids
Kone Paul et al. [48]	Reported 11 patients with Kawasaki disease with 7 had coronary artery dilatation and one has myocarditis with Kawasaki disease shock syndrome.	IVIG
Lind-Hoist et al. [49]	12-week-old infant with development of coronary artery aneurysms and pericarditis on day 10 after admission.	IVIG, infliximab, Corticosteroids
Maggio et al. [50]	9-month-old girl with parvovirus infection developed Kawasaki disease and 26 days after treatment for Kawasaki disease developed coronary artery aneurysms.	IVIG
Gambacorta et al. [51]	9-month-old boy with refractory classical Kawasaki disease who developed aneurysms.	IVIG, corticosteroids, infliximab

Kone-Paul et al. reported the use of anakinra in 11 patients. Nine of them had complete Kawasaki disease, and two of them had incomplete Kawasaki disease. They were started on anakinra with a dose range of 2-8mg/kg/day for 15 days after the deterioration of their clinical conditions despite treatment with primary (aspirin and IVIG) and secondary drugs (infliximab, cyclosporine, methotrexate) drugs. Complete resolution of fever and inflammation and reduction in the size of aneurysms were observed in these patients [48]. 

Lind-Hoist et al. reported atypical Kawasaki disease in a 12-week-old infant, treated with IVIG and aspirin. A refractory form of Kawasaki disease is diagnosed when symptoms worsen after treatment with infliximab and corticosteroids. Anakinra is administered when levels of ferritin, triglycerides, alanine aminotransferases, and hemophagocytosis in the bone marrow are observed and suspicious of macrophage activation syndrome is made and symptoms resolved with increasing doses from 5mg/kg/day to 10mg/kg/day [49].

Maggio et al. reported Kawasaki disease in siblings after being infected with parvovirus. The elder sibling died of coronary artery aneurysms due to Kawasaki disease, and after two years, the younger sibling at nine months of age developed incomplete Kawaski disease with subsequent coronary artery aneurysm on the 26th day of illness despite treatment with IVIG and administered anakinra with a dose of 4mg/kg/day for 25 days and reported improvement in her clinical conditions and reduction of aneurysm size [50]. Gambacorta et al. reported a case of classical Kawasaki disease in a nine-month-old boy refractory to treatment with IVIG on day nine and day 14, methylprednisolone on day 20, development of coronary artery aneurysms on day 37, and improvement in clinical condition and decrease in z score of aneurysms was observed after starting anakinra with the dosage of 6mg/kg/day on day 40 of illness [51].

Infliximab, TNF-α antagonist, and etanercept, when administered with intravenous immunoglobulins, cause rapid reductions in the level of c-reactive protein and resolution of fever in a few days and a decrease in z score of coronary artery aneurysms [52]. Etanercept, a TNF-α receptor blocker, when used as a primary drug with aspirin and intravenous immunoglobulins, reduces inflammation of blood vessels leads to resolution of fever, and the combination is well tolerated [53]. The involvement of the NFAT-calcineurin pathway in the pathogenesis of Kawasaki disease leads to the use of calcineurin inhibitors like cyclosporine and tacrolimus in the management of IVIG-resistant Kawasaki disease. These agents decrease the CD4+ T and CD8+T cell-mediated inflammation in blood vessels [54].

Coronary artery aneurysm risks stratification and follow-up strategies

**Figure 3:**
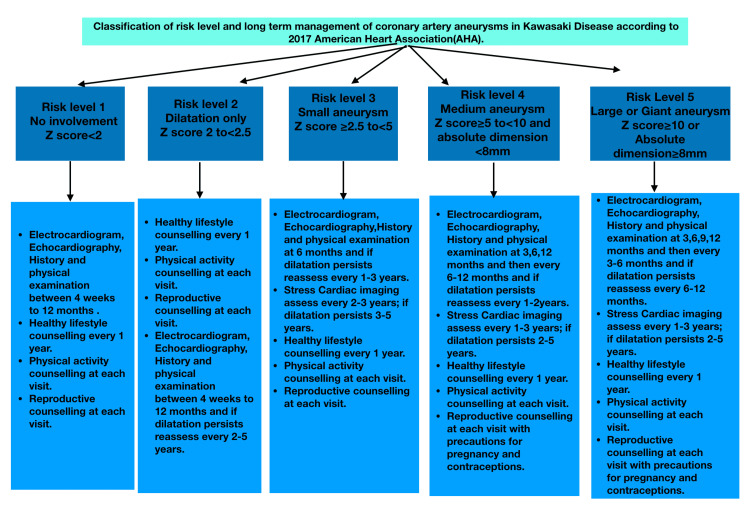
Follow protocol for coronary artery aneurysms in Kawasaki disease. Image Credits: Kruthiga Rajasekaran

Another important consideration in the treatment of Kawasaki disease is the prevention of cardiac complications. In 2017 The American Heart Association (AHA) proposed effective follow-up strategies for early detection and prevention of cardiac complications of Kawasaki disease (Figure 3).

Coronary artery aneurysms are one of the triggers for acute coronary syndrome as a complication of Kawasaki disease. Damage to vascular endothelium causes thrombus formation and can present as acute myocardial infarction. Patients should be monitored periodically with ECG, echocardiogram, and CT/MR angiography based on risk assessment to prevent this. Pharmacological interventions such as lipid-lowering drugs (statins) and anti-platelets like aspirin or clopidogrel should be advised [39].

Limitations

This is the generalized review of the pathogenesis, diagnosis, prevention, and treatment protocols for managing coronary artery lesions in Kawasaki disease. This article does not include the contraindications of drugs and patient-specific management of coronary artery lesions in Kawasaki disease. Therefore, each patient should be evaluated thoroughly and managed according to their history, clinical examination, and laboratory findings in clinical practice.

## Conclusions

Coronary artery lesions in Kawasaki disease are associated with significant morbidity and mortality. Although there are many interrelated mechanisms between genetic, immune, and infectious factors in the pathogenesis of coronary artery involvement in Kawasaki disease, the significant etiology has yet to be pinpointed. Echocardiogram, Harada scoring system, and biomarkers like NT-Pro-BNP help identify the risk of developing coronary artery aneurysms. The combination of aspirin with Intravenous immunoglobulins, corticosteroids, and biological agents like IL-1 antagonist reduces coronary consequences in Kawasaki disease. Although, as mentioned above, diagnostic and treatment strategies help prevent and treat coronary artery aneurysms, research should be conducted to find the etiology, gold-standard diagnostic tests, and pharmacological treatments on coronary artery lesions in Kawasaki disease.
